# Pain and sensory abnormalities in prodromal and early Parkinson's disease: mechanisms, assessment, and clinical implications

**DOI:** 10.3389/fpain.2026.1888196

**Published:** 2026-08-13

**Authors:** Maria Skallerup Andersen, Lorenz Martin Oppel, Boris Modrau, Kimmo Jensen, Parisa Gazerani

**Affiliations:** 1 Department of Neurology, Aalborg University Hospital, Aalborg, Denmark; 2 Department of Clinical Medicine, Aalborg University, Aalborg, Denmark; 3 Department of Life Sciences and Health, Faculty of Health Sciences, Oslo Metropolitan University, Oslo, Norway; 4 Department of Health Science and Technology, Faculty of Medicine, Aalborg University, Aalborg, Denmark

**Keywords:** drug-naïve Parkinson's disease, nerve conduction studies, pain, Parkinson's disease, prodromal Parkinson's disease, quantitative sensory testing, sensory abnormalities, small-fiber dysfunction

## Abstract

Parkinson's disease (PD) is traditionally characterized by motor symptoms; however, non-motor symptoms, particularly pain and sensory disturbances, are increasingly recognized as potential early manifestations that precede motor onset. Pain has been reported in up to 59% of patients before the onset of motor symptoms and in up to 72% in the early stages of PD, yet its underlying mechanisms and clinical implications remain insufficiently understood. Emerging evidence suggests that dopaminergic dysfunction may alter nociceptive processing across both peripheral and central pathways, involving changes within spinal, brainstem, and cortical pain networks. Despite this, the heterogeneity of pain phenotypes and the lack of standardized assessment approaches continue to limit the diagnostic and clinical utility of pain as an early disease indicator. Importantly, early pain symptoms may lead to misinterpretation and unnecessary investigations, potentially delaying PD diagnosis. This narrative review critically examines four key aspects of pain in prodromal to early-stage PD: (1) pain as an early clinical feature, (2) underlying nociceptive and neurodegenerative mechanisms, (3) alterations in small-fiber function assessed by quantitative sensory testing, and (4) large-fiber involvement evaluated through nerve conduction studies. By integrating current evidence, this review highlights the role of pain as a potential early indicator of PD, synthesizes current knowledge on sensory dysfunction across the prodromal to early disease spectrum, and identifies key gaps to support diagnosis, improved patient stratification, and mechanism-based management strategies.

## 1 Introduction

Over the past decade, pain has gained increasing attention as an important non-motor manifestation of prodromal, *de novo*, drug-naïve, and early-stage Parkinson's disease (PD) (1–7). Between 20%–59% of patients report pain prior to the onset of motor symptoms (3, 8). In early-stage PD, 55%–72% of patients experience chronic pain, contributing to reduced quality of life and impaired daily functioning (9–11). Pain is therefore among the most common and burdensome non-motor symptoms (NMS) (12), promoting increasing interest in its underlying mechanisms and pathophysiology. Pain in early-stage PD may arise partly from subtle, often undetected motor disturbances and, in some patients, may precede overt motor symptoms by several years (13). Pain is now recognized as a pre-motor feature of PD and may offer opportunities for earlier identification, improved diagnostic accuracy, and optimized early treatment strategies. Despite these advances, important questions remain regarding the mechanisms underlying pain, its temporal relationship with neurodegeneration, and its potential value as an early disease marker. Hence, further research is needed to better characterize clinical manifestations and underlying pain mechanisms in the early stages of PD.

Several classification systems have been developed to characterize pain in PD, including phenotype- and taxonomy-based classification frameworks (14, 15). More recently, the PD Pain Classification System (PD-PCS) has been proposed as a mechanism-based framework for characterizing PD-related pain (16, 17).

Quantitative sensory testing (QST) is widely used to evaluate somatosensory function and nociceptive processing by assessing responses to standardized mechanical, thermal, and other evoked stimuli. These approaches are widely used to evaluate alterations in nociceptive processing. Several studies have investigated nociceptive processing and sensory function in patients with PD to determine whether pain sensitivity differs from that of healthy controls. Most studies have focused on late-stage PD (disease duration ≥ 5 years). Studies in late-stage PD consistently demonstrate sensory abnormalities, including lower tactile thresholds, pain thresholds, and pain tolerance compared with healthy controls (18–21). Even though data from late-stage PD patients showed lower thresholds [e.g., electrical pain threshold (EPT), heat pain threshold (HPT), Nociceptive flexion reflex (NFR)] compared with controls, the difference in conditioned pain modulation did not reach statistical significance between late-stage PD patients and controls (20). Additionally, a non-significantly lower threshold (EPT, HPT, and NFR) was observed when comparing PD patients with pain to those without (20). Overall, sensory disturbances appear to become more pronounced with disease progression. In contrast, studies in prodromal and early-stage PD remain limited and report heterogeneous findings (Table 1).

**Table 1:** Summary of studies investigating pain and quantitative sensory testing (QST) in drug-naïve to mid-stage Parkinson's disease, ordered by disease duration.

Authors	Study population	Age (years ± SD) or Mean	Disease duration (years ± SD)	H&Y UPDRS-III (ON/OFF state)	OFF/ON state during pain testing	Method and Pain Measure	Stimulated area(s)	Main findings
*De novo* (no PD medication)								
Fründt et al. (5)	19 dnPD (8 PDP, 11 PDo) 19 HC	dnPD 64.8 ± 10.0 HC 64.8 ± 9.9	–	**H&Y:** Stage I: 4 dnPD Stage II: 12 dnPD Stage II.5: dnPD **UPDRS:** NA	OFF state	**Pain threshold**: CDT (C°), WDT (C°) CPT (C°), HPT (C°) PPT (kPa), MPT, MDT, VDT **Pain sensitivity**: MPS TSL (C°) WUR **Pain assessment**: Z-score	Hand	No significant abnormalities in QST parameters.
Early-stage (<5 years)								
Tinazzi et al. (50)	11 PDo (treated) 6 PDo (drug-naïve) 11 HC	PD treated 62 PD drug-naïve 64 HC 60	PD treated 3.5 PD drug-naïve 1.2	**H&Y:** NA **UPDRS:** NA	OFF state	**Pain threshold:** Scalp CO2 LEP (Watt) **Pain rating:** NRS	Foot	Reduced N2/P2 amplitudes and increased pain ratings were observed bilaterally in treated PD but only on the affected side in drug-naïve PD.
Mylius et al. (10)	14 PD 27 HC	PD 64.2 ± 8.1 HC 63.7 ± 11.54	1.8 ± 1.5	**H&Y:** 1.3 ± 0.5 **UPDRS (ON):** 19.8 ± 8.0	OFF state	**Pain threshold:** NFR (mA) HPT (C°) EPT (mA). **Pain rating:** VAS	Arm Leg	Reduced NFR threshold; unchanged HPT and EPT.
Boura et al. (9)	15 PD 27 HC	PD 64.5 ± 7.8 HC 63.7 ± 11.5	2.0 ± 1.6	**H&Y:** 1.3 ± 0.5 **UPDRS (ON):** 19.6 ± 7.8	OFF state	**Pain threshold:** NFR Threshold (mA) EPT (mA) HPT (C°). **Pain rating:** VAS	Arm	Reduced NFR threshold; no significant changes in EPT or HPT.
Mylius et al. (48)	9 PD 27 HC	PD 64.7 ± 6.5 HC 62.6 ± 10.1	3.1 ± 3.4	**H&Y:** 1.3 ± 0.5 **UPDRS (OFF):** 13.8 ± 5.0	OFF state	**Pain threshold:** NFR threshold(mA) EPT (mA) HPT (C°). **Pain rating:** VAS	Leg	No significant differences in HPT, EPT, or NFR threshold.
Zambito-Marsala et al. (42)	13 PDo 13 HC	PD 60.69 ± 11.66 HC 56.07 ± 11.86	3.69 ± 1.37	**H&Y (median):** 1 (range: 1–1.5) **UPDRS (OFF):** 22.7 ± 5.1	OFF state	**Pain threshold:** Scalp CO2 LEP (amplitude). **Pain rating:** NRS	Hand	Reduced N2/P2 amplitudes. No significant differences in N1/P1 amplitudes or LEP latencies. No significant side differences in N1/P1 or N2/P2 amplitudes or latencies. Increased pain ratings.
Petschow et al. (41)	13 PD 13 HC	PD 48.62 ± 5.85 HC 54.46 ± 9.60	4.46 ± 1.94	**H&Y:** 1.27 ± 0.39 **UPDRS:** 13.31 ± 4.44	OFF state	**Pain threshold:** WPT (C°) HPT (C°) **Pain intensity:** VAS	Arm Foot	No significant differences in WPT and HPT.
Skallerup et al. (49)	12 PD 12 HC	PD 68.67 ± 5.5 HC 67.5 ± 5.39	PD ≤ 5	**H&Y:** NA **UPDRS:** NA	ON state	**Pain threshold and tolerance:** PPT (kPa) CPT (C°, time) **Mechanical and Dynamic threshold:** Pinprick and Brush **Pain rating:** VAS	Arm Hand Lower back	Mechanical hypersensitivity characterized by dynamic mechanical allodynia, pinprick hyperalgesia, reduced PPT, and shorter CPT tolerance time. No significant differences in CPT pain ratings.
Mid-stage (4–6 years)								
Djaldetti et al. (51)	36 PD (21 PDP, 15 PDo) 28 HC	All 61.8 ± 11.2 (PDP 63.3 ± 12.3, PDo 60.4 ± 10.0) HC 58.9 ± 6.6	PDP 6.6 ± 5.4 PDo 4.3 ± 4.1	**H&Y:** 1–2.5 (6.6 ± 5.4) **UPDRS:** PDP 23.3 ± 15.2 PDo 21.2 ± 8.1	OFF state	**Sensory threshold:** TT (*Von Frey filaments*) WST (C°) **Pain threshold:** HPT (C°) **Pain intensity:** VAS	Hand	Reduced HPT, particularly in patients with pain; unchanged TT and WST.
Schestatsky et al. (52)	18 PD (9 PDP, 9 PDo) 9 HC	PDP 61.2 ± 6.6 PDo 59.0 ± 8.8 HC 58.9 ± 5.4	PDP 6.0 ± 4.1 PDo 5.4 ± 3.6	**H&Y:** 1–2.5 **UPDRS (OFF/ON mean):** PDP 19.1 ± 2.3 PDo: 17.1 ± 3.7	OFF state	**Pain threshold:** WPT and HPT (C°) LEP (amplitude) **Sensory threshold:** LPT **Pain intensity:** VAS	Hand	Reduced HPT and LPT and increased N2/P2 amplitudes in PD patients with pain; no differences in WPT.
Zambito et al. (21)	106 PD (66 PDP, 40 PDo) 51 HC	All 69.9 ± 8. (PDP 69.7 ± 27.2, PDo 70.2 ± 9.3) HC 68.7 ± 10	All PD 5.7 ± 5.6	**H&Y:** All PD: 2.1 ± 0.9 (PDP 2.3 ± 0.8, PDo 1.8 ± 0.0) **UPDRS (OFF):** All PD 23.5 ± 13.6 (PDP 26.4 ± 13.9 PDo 18.7 ± 11.6)	OFF state	**Pain tolerance and Pain threshold:** EPT (mA) **Sensory threshold:** TT (mA) **Pain intensity:** VAS	Hand Foot	Reduced pain threshold and pain tolerance; no significant changes in TT. No significant differences in pain threshold between PDP and PDo.

CDT, cold detection threshold; CHEP, contact heat evoked potential; CPT, cold pressor threshold; dnPD, *de novo* Parkinson's disease; EPT, electrical pain threshold; HC, Healthy controls; HPT, heat pain thresholds; H&Y, Hoehn and Yahr; LEP, laser evoked potentials; LPT, laser pinprick threshold; MDT, mechanical detection threshold; MPP, mechanical pain perception; MPS, mechanical pain sensitivity; MPT, mechanical pain threshold; mo., months; NFR, nociceptive flexion reflex; NA, Not available; NRS, numeric rating scale; ON state, On Parkinson's disease medication; OFF state, Withdrawal of Parkinson's disease medication; PD, Parkinson's disease; PDo, Parkinson's disease without pain; PDP, Parkinson's disease with pain; PPT, pressure pain threshold; TSL, thermal sensory limen; TT, tactile threshold; UPDRS, Unified Parkinson's Disease Rating Scale; VAS, visual analog scale; VDT, vibration detection threshold; WDT, warm detection threshold; WPT, warmth pain threshold/warmth perception thresholds; WST, warmth sensation threshold.

Peripheral neuropathy (PNP) has also been investigated using several complementary methodologies. Electrophysiological assessments of large nerve fibers have been used to investigate peripheral nervous system involvement and potential effects of levodopa exposure. Studies have reported that the prevalence of PNP ranges from 19 to 55% in late-stage PD (22–26). However, studies using nerve conduction velocity (NCV) in prodromal, *de novo* PD (dnPD), and early-stage PD are limited, which highlights the need for further neurophysiological investigations in the early stages of the disease.

This narrative review synthesizes current evidence on pain and sensory abnormalities across the disease spectrum from prodromal to early-stage PD (prodromal, dnPD, drug-naïve, and early-stage PD). The review focuses on four domains related to altered pain processing and modulation: (1) pain as an early clinical feature, (2) underlying nociceptive and neurodegenerative mechanisms, (3) alterations in small-fiber function assessed by quantitative sensory testing, and (4) large-fiber involvement evaluated through nerve conduction studies.

Sensory disturbances and pain patterns in these disease stages remain insufficiently understood. To our knowledge, this is the first review to integrate these four domains of pain processing and sensory function in early-stage PD. Insights from the prodromal stage may contribute to a better understanding of early pain-related changes in PD. By integrating these complementary domains, this review provides a disease-stage perspective on pain and sensory dysfunction in PD, with potential implications for earlier diagnosis, improved patient stratification, and future mechanism-based management strategies.

## 2 Methods

### 2.1 Search strategy

A literature search was conducted in PubMed in collaboration with the Medical Library at Aalborg University Hospital, covering publications from January 1995 to April 2026. The search strategy combined Medical Subject Headings (MeSH) and free-text terms applied to titles and abstracts, together with terms related to questionnaires and study design, and was restricted by language and publication date. The complete search strategy is provided in Supplementary Material 1.

Studies were included if they met the following criteria:

- Full-text, peer-reviewed articles
- Published in English
- Human observational clinical studies, including prospective, retrospective, cohort, case-control, and cross-sectional designs.
- Studies including participants with prodromal, *de novo*, drug-naïve, or early-stage PD (disease duration ≤ 5 years)
- Studies assessing pain, sensory abnormalities, nociceptive mechanisms, quantitative sensory testing (QST), or peripheral nerve involvement (Nerve conduction study).

Studies were excluded if they met any of the following criteria:

- Studies involving atypical parkinsonism
- Studies including PD patients with comorbid conditions likely to confound the results (e.g., psychiatric disorders, cancer, diabetes mellitus, substance abuse, or other central or peripheral neurological disorders)

*De novo* and drug-naïve PD patients were defined as individuals not previously exposed to PD-related medication. In some NCV studies, early-stage PD was defined as <3 years of PD medication use. Mid-stage PD (4–6 years disease duration) was defined according to studies comparing groups with shorter vs. longer disease duration. Two studies included patient groups with disease duration slightly exceeding 5 years.

Descriptive data, including participant characteristics, pain distribution, and prevalence of peripheral neuropathy, were extracted and summarized where available. Study selection and data extraction were performed by the authors based on predefined criteria. Due to substantial heterogeneity in study design, populations, and outcome measures, a qualitative narrative synthesis was performed, supplemented by descriptive summaries where appropriate.

## 3 Results and discussion

The search was completed in April 2026. A total of 2,262 records were screened, of which 28 articles met the inclusion criteria. An additional two articles were identified through backward citation searching. In total, 30 studies were included for data extraction.

This review included articles available on prodromal PD, dnPD, drug-naïve PD, and early-stage PD patients, including assessments of pain characteristics, pain classification, nociceptive mechanisms, QST, and PNP. Given the limited and heterogeneous evidence base, findings should be interpreted with caution, highlighting the need for further investigation.

### 3.1 Pain

#### 3.1.1 Is pain an initiating clinical feature in early-stage Parkinson's disease?

Gradually, pain has been recognized as an NMS that can manifest as one of the first symptoms of PD (11, 27–29). NMS, including pain, may emerge months to up to 10 years before the onset of overt motor symptoms (8, 11, 27, 29, 30). In some cases, pain may present more than 10 years prior to motor symptoms (8), supporting the notion that pain may represent an early clinical feature of PD. Walter et al. (31) identified musculoskeletal pain as one of the most common symptoms in the very early prediagnostic phase, appearing over 7 years before a PD diagnosis. Lin et al. (1) conducted a prospective study to investigate the relationship between preceding pain symptoms and the later development of PD by following 33,388 participants. Among them, 32 individuals developed PD during a mean follow-up period of 3 years. Participants reporting pain at baseline had a significantly higher likelihood of developing PD during follow-up compared to those without pain.

Pain, particularly musculoskeletal pain, is often present during the preclinical phases, years before the diagnosis of PD. Gonera et al. (29) found that patients with preclinical PD experienced more musculoskeletal pain, neurological-psychological, and cardiovascular symptoms compared to the control group. Among patients with preclinical PD, 52% reported musculoskeletal pain (29). Farnikova et al. (30) performed a retrospective study of 82 PD patients and found that musculoskeletal pain was present as a prodromal PD symptom in 33%. Walter et al. (31) conducted a retrospective study based on interviews with 115 PD patients regarding self-perceived prodromal non-motor features and found that 31.3% experienced prodromal pain approximately 5 years (5.2 ± 6.1) before diagnosis. Furthermore, 70% of PD patients reported musculoskeletal pain, although only 62% of these cases were determined to be PD-related at the time of diagnosis (31). One proposed explanation for early pain symptoms is reduced movement in larger joints on the PD-affected side, reflecting asymmetrical motor dysfunction due to bradykinesia and rigidity (2, 30).

Shoulder pain as an initial manifestation of PD has received increasing attention in recent years (2, 29). Collectively, retrospective studies consistently demonstrate that musculoskeletal pain, particularly shoulder pain, may precede the onset of motor symptoms by several years and is frequently misattributed to orthopedic conditions such as frozen shoulder, osteoarthritis, or degenerative spinal disease. Shoulder, neck, and back pain are among the most commonly reported prodromal pain locations, and registry-based studies further support an increased incidence of shoulder pain or stiffness before PD diagnosis. These observations suggest that shoulder pain may represent an important pre-diagnostic non-motor symptom that could facilitate earlier recognition of PD (2, 29–32).

In the early stages of PD, pain remains a significant factor affecting patients’ daily functioning. Valkovic et al. (33) found that 23% of early-stage PD patients reported experiencing one pain complaint, 38% reported two pain complaints, and 9% reported three pain complaints. Additionally, Li et al. (7) found that approximately 20% of early-stage PD patients experienced musculoskeletal pain at one site, while 80% reported pain at two or more sites (disease duration 4.52 ± 3.26 years). Furthermore, musculoskeletal pain in early-stage PD primarily affects the lower extremities (53%–78%) (7, 34), followed by the upper and lower back (13%–45%) (7, 30, 34), and the upper extremities (28%–30%) (7, 34). Specifically, patients reported pain in the pelvis and thighs (13%) (34), shoulders (8%–33%) (7, 30, 34), and smaller areas such as hands and wrists (24%), ankles and feet (17%) (7), neck (1%–15%) (7, 34) and head (3%–10%) (7, 34), highlighting the heterogeneous distribution of pain across body regions.

**Figure 1:**
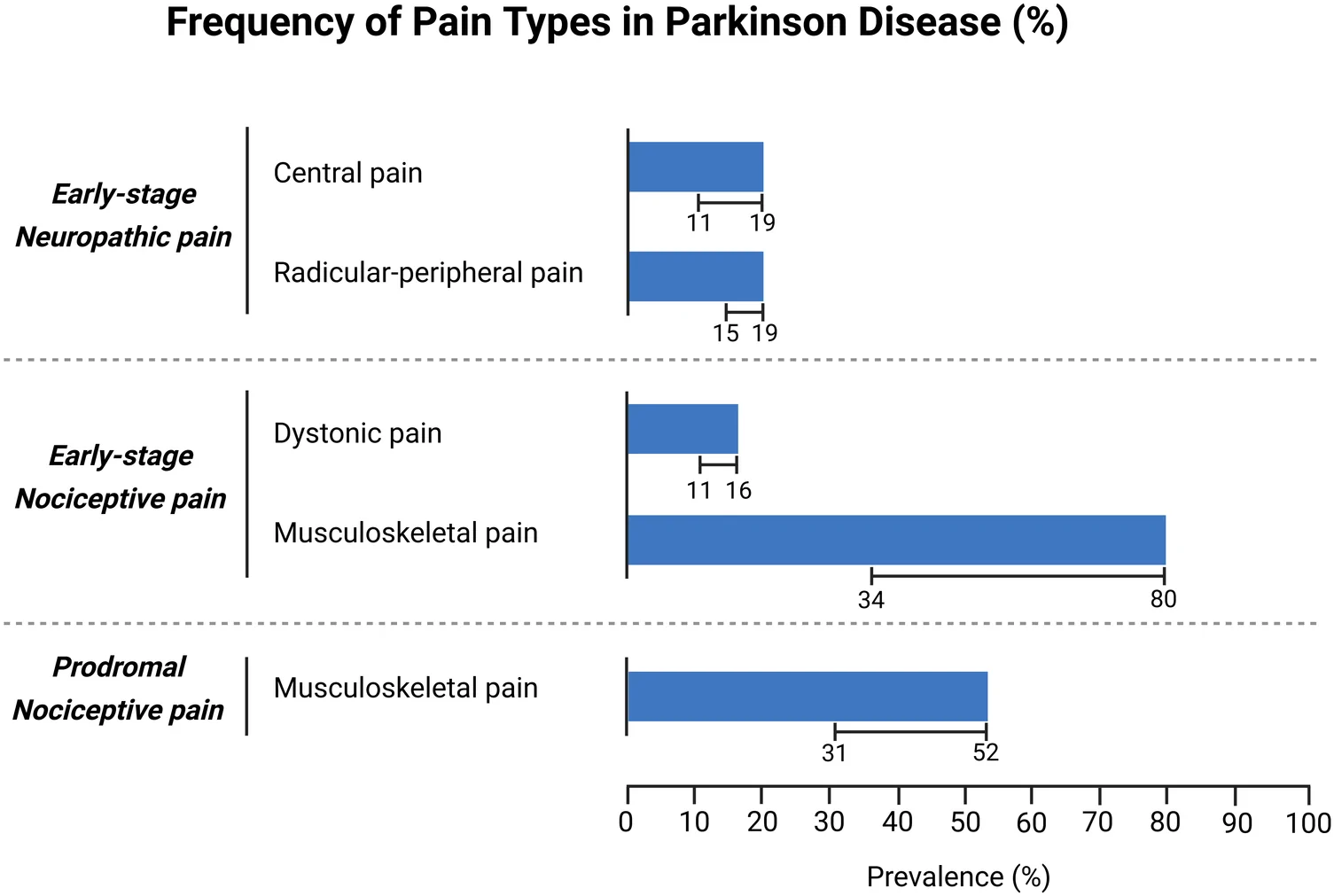
Frequency of different pain types in prodromal and early-stage Parkinson's disease. Pain categories are classified according to Ford et al. Central and radicular-peripheral pain are classified as neuropathic, whereas dystonic and musculoskeletal pain are nociceptive. The horizontal bars represent the reported prevalence ranges (minimum–maximum) for each pain type across the included studies. Gonera et al. (29), Farnikova et al. (30), Walter et al. (31), Valkovic et al. (33), and Fu et al. (34).

Pain in PD encompasses multiple types and is thought to arise from both nociceptive and neuropathic mechanisms. In PD, nociceptive pain is the most common and is associated with musculoskeletal and dystonic pain. Neuropathic pain can present as radicular-peripheral and/or central pain (Figure 1). Both nociceptive and neuropathic pain significantly impact the daily lives of patients with PD (35).

Based on Ford et al.'s (14) classification, Figure 1 illustrates the frequency of the first four pain types in PD patients across prodromal and early-stage (30, 31, 33, 34), akathisia is not classified as a pain type, but as a sensory complaint (35) and is beyond the scope of this review.

Several studies agree that musculoskeletal pain in prodromal and early-stage PD causes significant discomfort in daily living and thereby reduces the quality of life (7, 30, 34). Overall, musculoskeletal pain represents one of the most common pain complaints in prodromal and early-stage PD and may affect multiple body regions.

Over the past decade, several classification tools have been developed to characterize pain in PD. More recently, a consensus-based review by Tinazzi et al. (17) integrated previous PD pain classifications into a unified, mechanism-based framework. The review highlighted the PD-PCS as a tool that may address limitations of earlier systems by first distinguishing PD-related pain from unrelated pain and then classifying PD-related pain according to mechanistic descriptors (nociceptive, neuropathic, and nociplastic pain). This framework further supports mechanism-based diagnosis and may facilitate more targeted and individualized treatment strategies. Earlier classification systems contributing to this framework included several important tools. Ford et al. (14) introduced a widely used classification system comprising five pain categories in PD: musculoskeletal, dystonic, radicular-peripheral neuropathic, central neuropathic pain, and akathisia discomfort. Wasner and Deuschl et al. (15) subsequently proposed a taxonomy-based classification system, categorizing pain as nociceptive, neuropathic, and miscellaneous, with additional subcategories. However, due to the complexity of pain classification, more specific tools have been needed to validate pain in PD. Martinez-Martin et al. (36) later published the first validated patient-completed pain questionnaire specifically developed for PD. Mylius et al. (16) further developed the PD-PCS to distinguish PD-related pain from unrelated chronic pain and further subclassify PD-related pain into three groups based on validated mechanistic pain descriptors: nociceptive, neuropathic, and nociplastic pain, covering previously identified pain types in PD.

The observations above indicate that both motor and NMS may be present prior to a diagnosis of PD; however, it remains unclear whether motor or NMS initiates the disease process. While the temporal sequence remains unclear, available evidence suggests that subtle motor dysfunction may precede symptom awareness, whereas pain is often the first symptom perceived by patients. Sensory abnormalities appear to be frequent in PD but are often misdiagnosed or underdiagnosed, particularly in the prodromal and early stages.

Several studies suggest that pain may represent an early clinical feature of PD, occurring years before the onset of motor symptoms (8, 29–31). Walter et al. (31) further support this notion, reporting that 36% of prodromal PD patients consulted a physician due to NMS prior to diagnosis. Among these, pain was the most frequently reported symptom, with 20% of the total cohort seeking medical attention specifically due to pain. This discrepancy indicates that pain is likely underrecognized as an early manifestation of PD. Notably, musculoskeletal pain, particularly shoulder pain, is frequently misdiagnosed as orthopedic conditions, which may delay PD diagnosis and subsequent treatment, potentially negatively affecting quality of life (30, 32).

However, the reported prevalence and distribution of pain vary considerably across studies. This variability likely reflects differences in study design, patient populations, retrospective methodologies, and the lack of standardized pain definitions in PD. Retrospective assessments may introduce bias, which should be considered when interpreting these findings.

Within the PD-PCS framework, the predominance of musculoskeletal pain observed in prodromal and early-stage PD may reflect a primarily nociceptive mechanism, highlighting the value of mechanism-based classification in early disease stages and its potential to guide targeted treatment strategies.

Although the current classification (PD-PCS) defines three main pain types in PD, the possibility of additional, yet unidentified pain mechanisms cannot be excluded. Future studies may further refine and expand this classification, particularly in the prodromal and early stages of the disease.

In addition, patient-related factors may contribute to the heterogeneity of pain in PD. Previous studies, including patients in the early stages of PD, have reported a greater pain burden in women than in men (37–39). However, evidence specifically addressing gender-related differences in pain and sensory dysfunction during the early stages of PD remains limited.

In summary, these findings highlight the importance of recognizing pain, especially musculoskeletal pain, as a potential early feature of PD. Increased awareness among clinicians may improve earlier recognition of PD, particularly when pain occurs together with subtle motor signs such as bradykinesia. Overall, pain appears to be clinically relevant but often overlooked as an early indicator of PD and should be considered more carefully during the early diagnostic process.

#### 3.1.2 Pain pathology and nociceptive processing in early-stage Parkinson's disease

**Figure 2:**
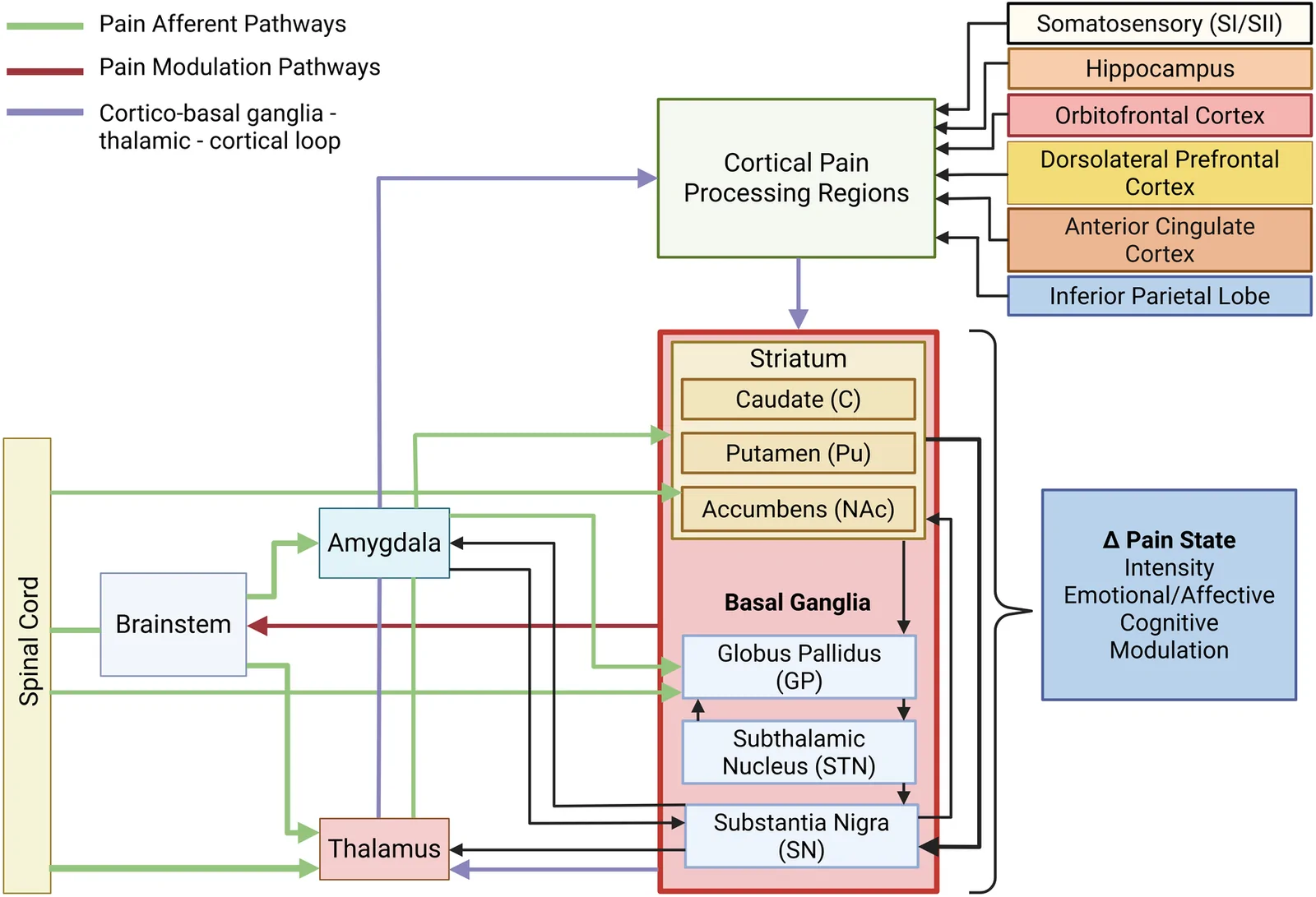
Basal ganglia involvement in pain processing and modulation. Schematic illustration of central nervous system structures involved in pain perception, nociceptive processing, and pain modulation. Afferent nociceptive input from the spinal cord and brainstem influences basal ganglia processing through direct and indirect interactions, with central integration involving the striatum and associated basal ganglia circuits. Thalamic projections to cortical pain-processing regions contribute to the cortico–basal ganglia–thalamic–cortical loop. Green arrows indicate afferent nociceptive pathways, red arrows represent modulatory pain-related pathways, and purple arrows illustrate cortico–basal ganglia–thalamic interactions. This figure is intended as a conceptual overview of basal ganglia-related pain networks rather than a complete neuroanatomical representation. C, caudate; GP, globus pallidus; NAc, nucleus accumbens; Pu, putamen; SN, substantia nigra; STN, subthalamic nucleus. Modified from Borsook et al. (2010) under the Creative Commons CC BY license.

Understanding of pain in PD has advanced; however, research has largely focused on neuronal degeneration and dopaminergic loss. PD should be considered a multisystem disorder, as NMS reflect widespread pathology and significantly impact quality of life (40). Pain remains a major clinical challenge, and the mechanisms underlying altered pain processing in PD are not yet fully understood. Degeneration within nociceptive pathways is complex and likely involves both peripheral and central structures at multiple levels (41, 42). Borsook et al. (43) proposed two major sources of nociceptive input to the basal ganglia: 1) ascending afferent pathways (e.g., spino-basal ganglia and spino-thalamic projections) and 2) cortical and subcortical inputs. Together, these pathways contribute to the cortico-basal ganglia–thalamic–cortical loop involved in pain processing (Figure 2). Building on this framework, Fil et al. (44) proposed that PD-related pathological changes within both peripheral and central nociceptive pathways may alter sensory processing at multiple levels, thereby affecting the interpretation and transmission of nociceptive information. Consequently, disrupted peripheral input together with altered cortical and subcortical processing may contribute to dysfunction of the basal ganglia pain network (43). In line with this concept, Truini et al. (45) suggested that abnormal basal ganglia function may modulate pain both directly, by influencing the transmission of nociceptive signals, and indirectly through affective and cognitive processes, thereby altering the perception and interpretation of pain. Furthermore, dopaminergic dysfunction within key nociceptive structures, including the spinal cord, thalamus, periaqueductal gray, basal ganglia, and cingulate cortex, may further contribute to altered pain processing in PD (44).

In summary, PD is characterized by dopaminergic dysfunction and should be considered a multisystem disorder, which complicates the identification of specific pain mechanisms. PD-related pathology may affect multiple regions within the pain-processing network.

Collectively, the available evidence suggests that early PD may involve alterations in peripheral sensory transduction and/or transmission to higher-order brain regions, leading to altered neuronal signaling and processing of sensory input, including pain. This interpretation is consistent with the mechanisms proposed by Fil et al. (44).

Based on the observations above, NMS, including pain and sensory disturbances, may overlap with motor features in prodromal and early PD, reflecting the multifocal nature of the disease.

These observations further support the involvement of both peripheral and central mechanisms in altered pain processing in PD. Standardized approaches to sensory assessment are therefore essential. QST provides insight into small-fiber function, whereas NCV studies assess large-fiber involvement. Together, these approaches provide complementary insights into peripheral sensory dysfunction in prodromal and early-stage PD. Understanding the interaction between peripheral and central mechanisms is essential for identifying early potential markers and developing mechanism-based interventions.

### 3.2 Sensory assessment of peripheral nerve function: quantitative sensory testing and nerve conduction velocity

To investigate peripheral sensory involvement in early-stage PD, complementary neurophysiological approaches are applied. QST provides a psychophysical assessment of somatosensory function, including modalities predominantly mediated by small fibers, whereas NCV evaluates large myelinated peripheral nerve function. Although NCV does not directly assess pain, both methods contribute to understanding sensory dysfunction and variability in pain presentation among PD patients.

Spontaneous pain is commonly assessed using instruments such as the visual analogue scale (VAS), numeric rating scale (NRS), and questionnaires including the McGill Pain Questionnaire and Parkinson's Disease Questionnaire-39. Evoked pain and underlying mechanisms have been assessed using QST, a widely applied method for evaluating small-fiber sensory function. QST encompasses multiple stimulus modalities, including mechanical (e.g., brush, pinprick, pressure), thermal (heat and cold), electrical, chemical (e.g., capsaicin), and laser-evoked potentials (LEPs) (46, 47).

However, findings remain inconsistent, and it is unclear whether patients across the early disease spectrum exhibit sensory disturbances such as allodynia or hyperalgesia. Consequently, the relationship between altered sensory processing and disease progression remains insufficiently understood.

To investigate potential changes in sensory abnormalities across disease stages, the available QST studies are presented according to disease stage. The following sections summarize findings in dnPD, early-stage PD, and mid-stage PD, followed by studies comparing the affected and less-affected sides.

#### 3.2.1 Pain and sensory assessment in *de novo* Parkinson's disease

QST findings in dnPD are summarized in Table 1 (5, 9, 10, 21, 41, 42, 48–52). Fründt et al. (5) assessed 13 somatosensory parameters in dnPD and found no significant differences between patients and healthy controls across all QST measures, including cold detection threshold, warmth detection threshold, thermal sensory limen, cold pain threshold (CPT), HPT, pressure pain threshold, mechanical pain threshold, mechanical pain sensitivity, wind-up ratio, mechanical detection threshold, and vibration detection threshold. Furthermore, no significant differences were observed between dnPD patients with chronic pain and those without chronic pain. All values were within normal reference ranges.

Fründt et al. (5) also investigated the affected and less affected body sides. Side-specific analysis showed reduced mechanical detection threshold and increased wind-up ratio on the affected side; however, these differences were not significant after Bonferroni correction.

Based on the currently available evidence from a single study, patients with dnPD do not appear to exhibit detectable alterations in sensory function compared to controls. However, subtle sensory abnormalities cannot be excluded, as changes at this early stage of the disease may be too small to detect. Observed side differences should therefore be interpreted with caution due to lack of statistical significance after correction. Overall, current evidence suggests that detectable small-fiber dysfunction may not yet be present in dnPD and that sensory abnormalities may become more pronounced with disease progression.

#### 3.2.2 Pain and sensory assessment in early-stage Parkinson's disease (treated with dopaminergic medication)

Most studies assessed patients in the OFF state, with only one study performing sensory testing in the ON state (Table 1) (49). Overall, studies in medicated patients with early-stage PD suggest subtle alterations in pain processing rather than a uniform pattern of sensory dysfunction. Evidence of mechanical hypersensitivity, including dynamic mechanical allodynia and pinprick hyperalgesia, was reported in one study (49). In contrast, LEP studies consistently demonstrated reduced N2/P2 amplitudes and increased pain ratings (42, 50). Findings from conventional QST remain inconsistent, with some studies reporting altered nociceptive reflex measures or pain thresholds (9, 10), whereas others found no significant differences in sensory thresholds or nociceptive reflex measures compared with healthy controls (9, 10, 41, 48). Detailed study characteristics and outcomes are presented in Table 1.

The variability in QST findings should be interpreted with caution. Methodological differences in QST protocols, outcome measures, stimulated body regions, patient populations, and disease severity may all contribute to the heterogeneous findings across studies. Furthermore, the heterogeneous findings may also reflect that neurodegenerative changes during the early stages of PD are not yet sufficiently advanced to produce consistent abnormalities across sensory modalities. Medication withdrawal before sensory assessment may better reflect disease-related mechanisms, but could also influence pain perception because of increased symptom burden. Consequently, further longitudinal studies using standardized sensory assessment protocols are needed to clarify the extent, temporal development, and clinical significance of pain and sensory abnormalities in early-stage PD.

#### 3.2.3 Pain and sensory assessment in mid-stage Parkinson's disease (4–6 years, treated with dopaminergic medication), with and without pain

All studies assessed patients in the OFF state (Table 1).

In contrast to early-stage PD, investigations of mid-stage PD primarily compared sensory function according to pain status. Overall, two studies reported greater sensitivity to painful stimuli in PD patients with pain than in patients without pain. These findings included lower HPT in both studies (51, 52), while one study additionally reported lower laser pinprick thresholds and increased LEP N2/P2 amplitudes (52). In contrast, one study found no significant differences in pain or tactile thresholds between PD patients with and without pain, although pain thresholds and pain tolerance remained lower in PD patients than in healthy controls (21). Detailed study characteristics and outcomes are presented in Table 1.

Overall, evidence regarding sensory differences between PD patients with and without pain remains inconclusive. While two studies reported greater sensitivity to painful stimuli and altered nociceptive processing in PD patients with pain (51, 52), these findings were not confirmed consistently across all studies (21). This inconsistency may reflect differences in patient selection, pain phenotypes, and sensory assessment protocols, but may also indicate that pain-related sensory abnormalities vary among patients with PD. The increased LEP N2/P2 amplitudes reported in one study may suggest altered central processing of nociceptive stimuli in PD patients with pain (52). The underlying mechanisms remain unclear. Further longitudinal studies using standardized sensory assessment protocols and well-defined pain phenotypes are needed to clarify the relationship between pain and sensory dysfunction in PD.

#### 3.2.4 Pain and sensory assessment of the affected vs. less-affected side in Parkinson's disease (drug-naïve to mid-stage, treated with dopaminergic medication)

All included studies were conducted in the OFF state (Table 1). Three studies investigated sensory lateralization in PD by comparing the affected and less-affected sides. Overall, findings were inconsistent. Increased sensitivity on the affected side was reported in PD patients with pain, whereas no side differences were observed in patients without pain (52). In drug-naïve PD, LEP abnormalities were confined to the affected side, whereas bilateral abnormalities were observed in treated PD (50). In contrast, no significant side differences in N2/P2 amplitudes were observed in another LEP study (42).

Based on the limited available evidence (42, 50, 52), it remains unclear whether sensory lateralization represents a consistent feature of PD. However, increased sensitivity on the affected side may reflect the asymmetric nature of PD, in which motor symptoms and dopaminergic neurodegeneration often begin unilaterally (53). The asymmetric nature of nigrostriatal degeneration in PD may contribute to side-related alterations in pain perception and nociceptive processing (53, 54).

Whether these alterations primarily reflect central or peripheral mechanisms remains unresolved and may vary between patients (54). Emerging models, such as the *α*-synuclein origin site and connectome (SOC) model, may provide future insight into these mechanisms (53, 55). Musculoskeletal factors, including rigidity, may also contribute to altered pain sensitivity in PD.

Overall, findings from QST and LEP studies suggest that sensory disturbances in PD may involve altered nociceptive processing, whereas evidence for side-related abnormalities remains inconsistent. As QST primarily assesses small-fiber mediated sensory function, NCV may provide complementary information regarding large-fiber peripheral nerve dysfunction.

### 3.3 Objective assessment with nerve conduction velocity of large peripheral nerve fibers in Parkinson's disease

Peripheral neuropathy (PNP) encompasses a range of disorders affecting the peripheral nervous system, with prevalence increasing with age (56). PNP is a common cause of chronic pain in the elderly (57). As PD predominantly affects older individuals, it remains unclear whether PNP arises from aging, PD-related mechanisms, levodopa exposure, or a combination of these factors. Recent studies indicate a greater prevalence of PNP among PD patients. Reported prevalence of PNP in dnPD varies widely (5%–67%) (26, 58, 59), compared with 8%–31% in elderly controls (22, 23, 25, 58, 60).

Alterations in vitamin metabolism (e.g., B12, homocysteine, methylmalonic acid) have been associated with PNP in PD and may relate to levodopa metabolism. Studies report lower B12 levels and higher homocysteine and methylmalonic acid levels in PD patients with PNP compared with patients without PNP and controls (22, 23, 25, 26). A detailed discussion is beyond the scope of this review.

PNP in PD has been assessed using electrophysiological methods (e.g., nerve conduction velocity, NCVs) alongside clinical scoring systems such as the Total Neuropathy Score (TNSr), the modified Toronto Clinical Neuropathy Score (mTCNS), and Utah Early Neuropathy Scale (UENS) (23, 26, 58, 60). Table 2 summarizes studies across different disease stages and medication states (26, 58–61).

**Table 2:** Summary of studies investigating peripheral neuropathy and nerve conduction abnormalities in *de novo* to mid-stage Parkinson's disease, ordered by disease duration.

Authors	Study population	Age (mean ± SD, years)	Disease duration (mean ± SD, years)	H&Y UPDRS-III (ON/OFF state)	Method	Area	Main findings
*De novo* (no exposure to Levodopa)							
Lee and Baik (59)	dnPD 105 (24 with PNP, 81 without PNP)	dnPD with PNP 74.1 ± 8.94 dnPD without PNP 67.2 ± 8.94	dnPD with PNP 0.9 ± 0.59 nPD without PNP 1.3 ± 1.33	**H&Y:** dnPD with PNP 1.5 ± 0.59 dnPD without PNP 1.5 ± 0.65 **UPDRS:** dnPD with PNP 22.6 ± 8.39 dnPD without PNP 22.9 ± 10.52	Electrophysiological assessment	**MNCS:** Peroneal nerve **SNCS:** Median nerve Ulnar nerve Sural nerve	PNP prevalence: dnPD 23%. Abnormal motor and sensory NCV findings.
Schindlbeck et al. (58)	39 dnPD 32 HC	dnPD 66.4 ± 12.3 HC 66.4 ± 12.4	1.75 ± 0.9	**H&Y**: 2.1 ± 0.6 **mUPDRS:** 27.4 ± 9.8	mTCNS, electrophysiological assessment	**MNCS:** Median nerve Ulnar nerve Peroneal nerve Tibial nerve **SNCS:** Median nerve Ulnar nerve Sural nerve	PNP prevalence: dnPD 67%, HC 31%. Increased mTCNS scores. No significant motor or sensory abnormalities.
Ceravolo et al. (26)	83 dnPD 137 HC	Age reported by sex and PNP status; no pooled mean reported.	3	**H&Y:** NA **UPDRS:** 15.7	TNSr, electrophysiological assessment	**MNCS:** Peroneal nerve **SNCS:** Sural nerve	PNP prevalence: dnPD 5%, HC 9%. Increased TNSr scores. Predominantly sensory axonal neuropathy.
Early-stage (exposed to Levodopa or other types of PD medication <3 years)							
Podgorny et al. (60)	26 Predominantly levodopa-naïve PD 22 HC without PNP	PD 63 HC 63	0.92 (0.01–4.02)	**H&Y:** NA **UPDRS:** NA	UENS, electrophysiological assessment	**SNCS:** Superficial peroneal nerve Sural nerve	PNP prevalence: PD 38%, HC 23%. No significant NCV or overall UENS differences. Subtle trends toward reduced sensory nerve function.
Ceravolo et al. (26)	103 short-term PD 137 HC	Age reported by sex and PNP status; no pooled mean reported	3.8	**H&Y:** NA **UPDRS:** 18.2	TNSr, electrophysiological assessment	**MNCS:** Peroneal nerve **SNCS:** Sural nerve	PNP prevalence: Short-term PD 7%, HC 9%. Increased TNSr scores. Predominantly sensory axonal neuropathy.
Rajabally and Martey (61)	33 predominantly levodopa-naïve PD	PD 61.8 ± 8.15	3.09 ± 2.20	**H&Y:** NA **UPDRS:** NA	UENS in all patients, electrophysiological assessments performed only in a subset of patients	**MNCS:** NA **SNCS:** NA	PNP prevalence: PD 12% *PNP identified primarily by UENS*

dnPD, *de novo* Parkinson's disease; HC, Healthy control; H&Y, Hoehn and Yahr scale; mTCNS, modified Toronto Clinical Neuropathy Score; MNCS, Motor nerve conduction studies; NA, Not available; NCV, Nerve conduction velocity; PD, Parkinson's disease; PNP, Peripheral neuropathy; SNCS, Sensory nerve conduction; TNSr, Total neuropathy score; UPDRS, Unified Parkinson's Disease Rating Scale; UENS, Utah Early Neuropathy Scale.

#### 3.3.1 Peripheral neuropathy in *de novo* Parkinson's disease (no exposure to levodopa)

The prevalence of PNP in dnPD varied substantially across studies ranging from 5% to 67%, with prevalences in healthy controls ranging from 9% to 31%, where control groups were included (26, 58, 59). Notably, PNP was observed in dnPD patients despite the absence of dopaminergic medication exposure. Detailed study characteristics and outcomes are presented in Table 2.

Across studies, neuropathy was assessed using combinations of NCV and clinical neuropathy scoring systems (TNSr and mTCNS), with disease duration ranging from approximately 9 months to 3 years (26, 58, 59). However, findings were inconsistent, with abnormalities reported in both clinical neuropathy scores and electrophysiological assessments, although these findings were not consistently observed across studies (26, 58, 59).

Overall, PNP prevalence in dnPD ranges from similar to controls to markedly higher, despite the absence of PD medication exposure. However, the limited number of available studies restricts conclusions regarding whether PD contributes to the development or progression of PNP.

Across studies, neuropathy assessments were often limited to selected nerve fibers and did not include a comprehensive neurophysiological evaluation, limiting the ability to determine the extent and pattern of nerve involvement in dnPD. Furthermore, some studies reported combined sensory and motor involvement (26, 59), while others reported predominantly sensory, axonal neuropathy (26).

Overall, current evidence does not allow firm conclusions regarding the presence or nature of PNP in dnPD. It remains unclear whether the observed findings reflect PD-related mechanisms or methodological differences, including partial NCV assessments and variability in diagnostic criteria, which further limit comparability between studies. These findings highlight the need for standardized and comprehensive neurophysiological assessments in early PD populations.

#### 3.3.2 Peripheral neuropathy in early-stage Parkinson's disease (exposed to levodopa or other types of Parkinson's disease medication in daily use <3 years)

Evidence on PNP in early-stage PD patients with minimal or recent exposure to dopaminergic medication remains limited. The included studies comprised mixed populations of predominantly levodopa-naïve patients and patients with limited exposure to dopaminergic medication, complicating interpretation (26, 60, 61).

Reported PNP prevalence ranged from 7% to 38% across studies. Compared with healthy controls, findings ranged from similar to slightly higher prevalences in patients with PD (26, 60, 61). Notably, PNP was observed both in predominantly levodopa-naïve patients and in patients with limited dopaminergic exposure. Detailed study characteristics and outcomes are presented in Table 2.

Across studies, peripheral nerve function was assessed using NCV alongside clinical neuropathy scoring systems. However, findings were inconsistent. While one study reported increased neuropathy scores together with abnormal NCV findings (26), another found no significant differences in NCV or overall UENS scores despite subtle trends toward reduced sensory nerve function (60), whereas a third identified PNP primarily using UENS (61).

In summary, it remains unclear whether PNP in PD is driven by disease-related mechanisms, dopaminergic treatment, metabolic factors, or a combination of these. However, evidence from dnPD and early-stage PD suggests that PNP may occur even in patients with minimal or no exposure to PD medication, supporting the possibility that neuropathy may, at least in part, develop independent of pharmacological treatment.

Reported PNP prevalence varies substantially across studies (7%–38%) (26, 60, 61), likely reflecting differences in study design, patient selection, and diagnostic criteria, which limit direct comparability.

The limited neurophysiological assessments and variation in the nerves examined make it difficult to determine which peripheral nerves are affected and the pattern of nerve involvement in early-stage PD. NCV investigations were often restricted to selected lower-extremity nerves (26, 60), while one study did not report which nerves were examined (61). Although some studies reported findings consistent with early axonal involvement (26, 61), current evidence remains insufficient to determine which nerve fibers are predominantly affected during the early-stages of PD.

Further studies using standardized and comprehensive neurophysiological assessments are needed to clarify the extent and pattern of peripheral nerve involvement in early-stage PD, as well as the relative contributions of PD-related and treatment-related mechanisms.

Overall, available NCV studies in dnPD and early-stage PD provide limited and heterogeneous evidence of peripheral nerve involvement. When neuropathy was identified, findings were generally consistent with axonal involvement, although mixed sensory and motor abnormalities were also reported. The occurrence of PNP in dnPD and early-stage PD suggests that neuropathy cannot be explained solely by dopaminergic treatment. However, the limited number of studies and methodological heterogeneity preclude firm conclusions regarding the type of PNP and its underlying mechanisms in the early stages of PD.

### 3.4 Integrated synthesis of pain and sensory dysfunction across disease stages in Parkinson's disease

The available evidence suggests that pain and sensory dysfunction may be present across the early stages of PD, although the clinical manifestations and findings from QST and NCV studies differ according to disease stage.

In the prodromal stage, pain, particularly musculoskeletal pain, appears to be a frequent NMS and may precede the onset of motor symptoms by several years (8, 29–32). Shoulder, neck, and back pain are among the most commonly reported complaints and may initially be attributed to musculoskeletal conditions such as frozen shoulder, osteoarthritis, or degenerative spinal disease (2, 29–32). However, no studies using QST or NCV were identified in prodromal PD, and the underlying sensory mechanisms therefore remain unclear at this stage.

In dnPD, available evidence suggests that pain may already be present at the time of diagnosis. However, the currently available QST evidence is limited to a single study, which reported no significant sensory abnormalities compared with healthy controls (5). These findings suggest that detectable small-fiber dysfunction may not yet be present in dnPD, although subtle sensory abnormalities cannot be excluded. Similarly, findings regarding PNP in dnPD remain inconsistent across NCV studies (26, 58, 59). Collectively, these findings suggest that while pain may already be clinically relevant in dnPD, evidence for sensory dysfunction remains limited and inconsistent.

In early-stage PD, pain is increasingly recognized as a clinically relevant symptom affecting daily functioning and quality of life (7, 33, 34). QST studies report heterogeneous findings, with some investigations demonstrating hyperalgesia, mechanical allodynia, altered nociceptive processing, and increased sensitivity to evoked pain compared with healthy controls (9, 42, 49, 50), whereas others report no detectable sensory abnormalities (10, 41, 48). NCV studies similarly provide inconsistent evidence of peripheral nerve involvement in early-stage PD, with findings ranging from no significant electrophysiological abnormalities to changes consistent with early axonal neuropathy (26, 60, 61). Taken together, these findings indicate that both peripheral and central mechanisms may contribute to altered sensory processing in early-stage PD. However, methodological heterogeneity and the limited number of studies preclude firm conclusions regarding the extent and temporal development of these abnormalities.

In mid-stage PD, available QST studies suggest alterations in nociceptive processing, particularly among PD patients experiencing pain (21, 51, 52). Lower pain thresholds and increased LEP amplitudes may indicate altered nociceptive processing and increased sensitivity to painful stimuli in PD patients with pain (51, 52). However, evidence regarding sensory differences between PD patients with and without pain remains inconclusive.

Interestingly, LEP studies reported reduced N2/P2 amplitudes in early-stage PD, whereas increased amplitudes were observed in mid-stage PD patients with pain, suggesting that cortical pain processing may vary according to disease stage and pain status.

Overall, the available literature suggests that pain may represent one of the earliest clinically detectable manifestations of PD (8, 29–32). While pain is frequently reported in prodromal and early-stage PD, findings from QST and NCV studies are more heterogeneous (5, 9, 10, 21, 26, 41, 42, 48–52, 58–61). The variability in these may reflect methodological differences between studies, limited sample sizes, and differences in study populations. Importantly, the available evidence suggests that pain may be clinically detectable before consistent abnormalities are identified using QST and NCV. Collectively, these findings support the concept that pain and sensory dysfunction may evolve differently during the early stages of PD, although their temporal relationship remains incompletely understood.

Although the present review focuses on pain characteristics and sensory dysfunction in prodromal to early-stage PD rather than treatment efficacy, these findings may have important clinical implications. The PD-PCS provides a framework for distinguishing PD-related from unrelated pain and for characterizing pain according to nociceptive, neuropathic, or nociplastic mechanisms, which may support more individualized clinical management (16, 17). Pharmacological treatment may include optimization of dopaminergic therapy and should be tailored to the predominant pain mechanism and relevant comorbidities (4, 16, 17). Non-pharmacological strategies, including individually adapted exercise, physiotherapy, psychological interventions, and multidisciplinary rehabilitation, may address mobility, function, coping, and psychosocial contributors to pain (62, 63). These approaches may therefore have complementary roles. However, direct evidence regarding the efficacy of either pharmacological or non-pharmacological pain interventions specifically in drug-naïve and early-stage PD remains limited.

Beyond pharmacological and non-pharmacological management, pain in PD should also be considered in relation to coexisting neuropsychiatric symptoms (17). Depression and anxiety frequently coexist with pain and may influence pain intensity, pain-related distress, functional impact, and symptom reporting (17, 64). Conversely, persistent pain may contribute to psychological distress and reduced quality of life (65). This relationship is likely bidirectional and may reflect overlapping neurobiological, psychological, and social mechanisms (17, 65). Mood symptoms should therefore be considered as part of a comprehensive pain assessment, including during the early stages of PD (17). However, evidence specifically investigating these relationships in drug-naïve and early-stage PD remains limited (17).

## 4 Limitations

This narrative review has several limitations that should be considered when interpreting the findings.

First, a key limitation of the current literature is the limited evidence available in prodromal, dnPD, drug-naïve, and early-stage PD. This limitation can be viewed from several perspectives: (a) the overall number of studies investigating pain, sensory abnormalities assessed by QST, and PNP assessed by NCV remains limited, particularly in prodromal, dnPD, and drug-naïve PD; (b) substantial methodological heterogeneity exists across studies, limiting direct comparisons between findings; and (c) primary research investigating pain-related pathology in early PD remains scarce, with current knowledge largely derived from theoretical and review-based literature. Direct investigation of pathology in the early stages is further complicated by the fact that pathological changes are primarily assessed post-mortem, limiting insight into the underlying mechanisms during the earliest phases of the disease.

Second, interpretation of QST findings is complicated by variability in medication states (ON vs. OFF), as residual effects of dopaminergic treatment may influence sensory responses. Additionally, medication withdrawal may affect physiological and subjective responses. This may affect the reported findings of increased or normal sensitivity to QST. As dopamine agonists have a long half-life (1–65 h) (66), and the central activity and clinical benefit of Levodopa may last up to 8 days (5, 67), remaining effects of treatment should be taken into account. Furthermore, medication withdrawal may influence physiological responses and patient-reported outcomes, thereby affecting the interpretation of QST findings.

Third, NCV findings are limited by methodological variability, including differences in the number and type of nerves assessed.

These limitations highlight the need for standardized methodologies, harmonized study designs, and greater emphasis on prospective investigations in early-stage PD.

## 5 Future directions/perspective

PD is a multifocal disorder with pathology extending beyond the central nervous system. Concepts such as Braak's hypothesis and the SOC model (brain-first vs. body-first) highlight the complex and heterogeneous nature of disease progression. NMS, including pain, may involve multiple systems and complicate early diagnosis and treatment. Future research should adopt multimodal and longitudinal approaches integrating pain characterization, QST, NCV, neuroimaging, sensory evoked potentials, and relevant biomarkers to improve understanding of pain mechanisms and facilitate earlier disease detection. Prospective longitudinal studies assessing patients from diagnosis through disease progression are needed to clarify temporal changes in sensory function and pain processing.

Key priorities for future research include: (1) systematic mapping of pain phenotypes to support earlier diagnosis, (2) standardization of QST protocols, including medication washout procedures, (3) harmonization of NCV methodologies, (4) development of non-invasive approaches to assess sensory dysfunction (e.g., evoked potentials), and (5) comparative evaluation of existing pain classification systems to identify optimal frameworks for PD research.

## 6 Conclusion

Improved recognition of early pain features may contribute to earlier diagnosis and more targeted management strategies in PD.

This review suggests that sensory abnormalities and altered pain processing may be present from the prodromal to early stages of PD, including in patients without exposure to dopaminergic treatment. Pain, particularly musculoskeletal pain, appears common in the early stages of PD and may initially be underrecognized or misattributed to other conditions, potentially delaying diagnosis. Notably, shoulder pain may represent one of the earliest clinically detectable manifestations of PD, as multiple retrospective studies have reported its occurrence several years before the onset of overt motor symptoms. While subtle motor dysfunction may precede clinical recognition, patients may first become aware of NMS, including pain and sensory disturbances. Greater awareness of pain as a potential early manifestation of PD may facilitate earlier recognition of the disease, particularly when pain occurs together with subtle motor signs or other prodromal features.

Findings from QST and NCV further suggest that both small-fiber sensory dysfunction and large-fiber peripheral nerve involvement may occur in some patients during the early stages of PD, although results remain heterogeneous. Overall, the available evidence supports the growing recognition of pain as an important early feature of PD. Improved recognition and characterization of early pain and sensory features may contribute to improved phenotyping and more individualized management strategies in PD.
