# Supplementary material for: Pain and sensory abnormalities in prodromal and early Parkinson's disease: mechanisms, assessment, and clinical implications
Source: Front Pain Res (Lausanne). 2026 Aug 13;7:1888196. doi: 10.3389/fpain.2026.1888196 (PMC13527717; doi:10.3389/fpain.2026.1888196)
Supplement: Supplementary file 1 [file Table1.docx]

# **Supplementary material**

**Appendix 1. search string**

| **PUBMED** | |
| --- | --- |
| #1 | ("Surveys and Questionnaires"[MeSH Terms] OR ("Epidemiologic Studies"[MeSH Terms] OR "cohort*"[Title/Abstract] OR "control"[Title/Abstract] OR "controls"[Title/Abstract] OR "interview*"[Title/Abstract] OR "questionnair*"[Title/Abstract] OR "cross sectional"[Title/Abstract])) AND (("Parkinson Disease"[MeSH Terms] OR "parkinson*"[Title]) AND ("Pain"[Title] OR "pains"[Title] OR "somatosensor*"[Title] OR "neuropath*"[Title] OR "nocicepti*"[Title] OR "musculoskeletal*"[Title] OR "non motor*"[Title] OR "nonmotor*"[Title] OR ("Nociceptive Pain"[MeSH Terms] OR "Pain"[MeSH Terms] OR "Nociception"[MeSH Terms] OR ("Musculoskeletal Pain"[MeSH Terms] OR "Musculoskeletal Diseases"[MeSH Terms]) OR "Sensory Thresholds"[MeSH Terms] OR ("Pain Management"[MeSH Terms] OR "Chronic Pain"[MeSH Terms] OR "Pain Perception"[MeSH Terms]) OR "Pain Measurement"[MeSH Terms] OR "Prodromal Symptoms"[MeSH Terms]))) AND ("english"[Language] AND 1995/01/01:2026/04/23[Date - Publication]) |
